# Multiple social platforms reveal actionable signals for software vulnerability awareness: A study of GitHub, Twitter and Reddit

**DOI:** 10.1371/journal.pone.0230250

**Published:** 2020-03-24

**Authors:** Prasha Shrestha, Arun Sathanur, Suraj Maharjan, Emily Saldanha, Dustin Arendt, Svitlana Volkova

**Affiliations:** 1 Data Sciences and Analytics, Pacific Northwest National Laboratory, Richland, WA, United States of America; 2 Physical and Computational Sciences, Pacific Northwest National Laboratory, Richland, WA, United States of America; 3 Visual Analytics, Pacific Northwest National Laboratory, Richland, WA, United States of America; Universitat de Barcelona, SPAIN

## Abstract

The awareness about software vulnerabilities is crucial to ensure effective cybersecurity practices, the development of high-quality software, and, ultimately, national security. This awareness can be better understood by studying the spread, structure and evolution of software vulnerability discussions across online communities. This work is the first to evaluate and contrast how discussions about software vulnerabilities spread on three social platforms—Twitter, GitHub, and Reddit. Moreover, we measure how user-level e.g., bot or not, and content-level characteristics e.g., vulnerability severity, post subjectivity, targeted operating systems as well as social network topology influence the rate of vulnerability discussion spread. To lay the groundwork, we present a novel fundamental framework for measuring information spread in multiple social platforms that identifies spread mechanisms and observables, units of information, and groups of measurements. We then contrast topologies for three social networks and analyze the effect of the network structure on the way discussions about vulnerabilities spread. We measure the scale and speed of the discussion spread to understand how far and how wide they go, how many users participate, and the duration of their spread. To demonstrate the awareness of more impactful vulnerabilities, a subset of our analysis focuses on vulnerabilities targeted during recent major cyber-attacks and those exploited by advanced persistent threat groups. One of our major findings is that most discussions start on GitHub not only before Twitter and Reddit, but even before a vulnerability is officially published. The severity of a vulnerability contributes to how much it spreads, especially on Twitter. Highly severe vulnerabilities have significantly deeper, broader and more viral discussion threads. When analyzing vulnerabilities in software products we found that different flavors of Linux received the highest discussion volume. We also observe that Twitter discussions started by humans have larger size, breadth, depth, adoption rate, lifetime, and structural virality compared to those started by bots. On Reddit, discussion threads of positive posts are larger, wider, and deeper than negative or neutral posts. We also found that all three networks have high modularity that encourages spread. However, the spread on GitHub is different from other networks, because GitHub is more dense, has stronger community structure and assortativity that enhances information diffusion. We anticipate the results of our analysis to not only increase the understanding of software vulnerability awareness but also inform the existing and new analytical frameworks for simulating information spread e.g., disinformation across multiple social environments online.

## 1 Introduction

Modern software practice is absolutely dependent on open source libraries and components. According to the 2018 Open Source Security and Risk Analysis report [1], open source components are now present in 96% of commercial codebases with an average of 257 different components per application. However, 78% of the codebases contained at least one vulnerability with an average of 64 vulnerabilities per codebase.

The National Vulnerability Database (NVD) [2] that curates and publicly releases vulnerabilities known as Common Vulnerabilities and Exposures (CVEs) is drastically growing and includes more than 100,000 known vulnerabilities to date. Some *vulnerabilities from NVD were successfully exploited by Advanced Persistent Threat (APT) groups (including APT groups associated with Russia) to execute major cyber attacks in 2017*, including but not limited to the NotPetya [3], Petya [4], and WannaCry [5] attacks. To reflect on the damages associated with these cyber attacks, the WannaCry attack in May 2017 involved over 200,000 victims, more than 300,000 computers, and caused 4 billion dollars in damages. In June 2017, NotPetya and Petya attacks have clearly shown that the functioning of an entire country (Ukraine) can be severely disrupted. These attacks happened because there were known vulnerabilities present in modern software and some APT groups effectively exploited them to execute a cyber attack.

In this work, we show the benefits of using social media to monitor discussions about software vulnerabilities and analyze their spread across social platforms. Along with their importance for national security [6, 7], studying the diffusion of discussions about software vulnerabilities is also useful to lay the foundation for cross-platform spread of information. Software vulnerabilities come in the form of a very identifiable and traceable piece of information, namely CVE IDs. These CVE IDs can help draw a clearer picture of how a particular piece of information can spread unlike narratives (that are extremely hard to identify), images (for which it is hard to measure similarity at scale) or URLs (that have to be un-shortened) whose diffusion paths are hard or sometimes even impossibles to trace. Similar to cybersecurity attacks [8, 9], information about software vulnerabilities is public and actively discussed online, for example, on cybersecurity-related websites and blogs and major social media platforms [10–12]. Interestingly, we found that there are more mentions of software vulnerabilities on Twitter and Reddit, compared to specialized platforms frequently used by core developer communities—StackOverflow and StackExchange. We found around 400 posts that mention CVE IDs in 24M StackOverflow answers and 15.5M questions. Out of 130,984 StackExchange posts, only 609 included mentions of non-unique CVE IDs.

**Fig 1:**
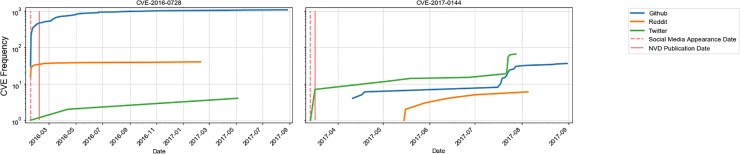
Example CVEs that appear on social media before published in NVD.

Moreover, we found evidence that social media can be used to monitor vulnerabilities before they are announced in official databases e.g., NVD. We estimated that nearly *a quarter of CVEs discussed in social media between 2015 and 2017 emerge on social platforms prior to their addition to the NVD database*. For these CVEs, the difference between the date when a CVE appears in one of the social platforms we studied and the date of its publication to NVD is 87 days on average. For example, in Fig 1 we present a vulnerability CVE-2017-0144 that was exploited during WannaCry and NotPetya cyber attacks in 2017, that is discussed on Twitter three days before it is added to NVD. Another vulnerability known to be exploited by Russian APT groups CVE-2016-4117 appears on all three platforms—Twitter, Reddit and GitHub on the same day a month before its announcement on NVD. Such trends of social media signals preceding official sources could potentially allow institutions to anticipate and prioritize which vulnerabilities to address first. Furthermore, quantification of the awareness of vulnerabilities and patches spreading in online social environments can provide an additional signal for institutions to utilize in their open source risk-reward decision making.

Our work takes the first steps towards this solution, which includes the following research questions that also summarize our major research contributions.

- Is there actionable software vulnerability signal in social media? Is this signal equally useful across multiple social platforms?
- What is the difference in network structure across social platforms for those users engaged in software vulnerability discussions, and how the network topology effect the way CVE discussions spread?
- How user-level or content-level characteristics of vulnerabilities e.g., severity, being targeted by a specific APT group, subjectivity of the post, vulnerabilities targeting a specific operating system or a product being spread by a bot vs. a human etc. change the rate of spread?

Unlike any previous work, this paper is the first to perform a contrastive analysis of discussion thread structure and growth in multiple social platforms. We start by presenting a multi-platform information spread framework in Section 2 and provide definitions for the unit of information, information spread criteria, mechanisms, observables, and measurements. Section 3 presents our data collection strategy for the domain of interest—software vulnerabilities for GitHub, Twitter, and Reddit. In Section 4, we perform an in-depth investigation into social network topology related to vulnerability discussions in three social platforms, and elaborate on how the network structure effects the CVE spread in each of the platform. We then quantitatively measure and contrast the speed and scale of discussion threads for vulnerabilities of varied severities in Section 5.3 and vulnerabilities targeted by different APT groups—North Korea, Russia, China, and Iran in Section 5.4 In Section 5.5 we see how information about different software products, in particular, operating systems spread. Finally, inspired by earlier work [13, 14], we evaluate information spread by conditioning on user-level and post-level factors, by contrasting the spread of vulnerabilities in discussion threads started by accounts predicted to be bots versus humans on Twitter, as well as discussions that start with posts predicted to be subjective (positive, negative) versus neutral on Reddit in Sections 5.6 and 5.7. In all our comparisons, we perform statistical significance testing and find that most of the differences that we found in discussion diffusion are significant.

## 2 Framework to measure multi-platform information spread

### Information

In order to study multi-platform information spread, we first must define what is meant by *the unit of information*, especially in the context of multiple social environments. In this context, information may consist of a specific piece of *knowledge*, such as a stated fact, opinion, or narrative; a specific piece of *content*, such as a tweet, post, webpage, image, or video; a specific *entity* such a person or event; or a specific practice or *behavior*, such as the use of different technologies or the adoption of different social norms.

For the purposes of this study, we focus on a set of specific software vulnerabilities as the units of information of interest, which fall into the entity category. This application area is particularly amenable to keyword-based tracking of information because each software vulnerability can be uniquely identified by a distinctive identifier specified by the National Vulnerability Database.

### 2.1 Information spread

Given a specified unit of information, we next want to measure its spread among users in multiple social environments. We want to ask “has the information spread?” (criteria), “how was the information spread?” (mechanism), “how do we know that the information has spread?” (observable), and “what are the characteristics of the spread?” (measure).

#### 2.1.1 Criteria

There are several criteria that could indicate the spread of the information from one user to another ranging from less to more active. Information can be considered to spread to a new user if that user observes the information (awareness), interacts with the information (engagement), endorses the information (approval), rebroadcasts the information (propagation), or changes their behavior in response to the information (adoption). The appropriate criteria for information spread depends partly on the specific type of information being studied, with the spread of specific facts or opinions being best studied under the lens of approval or propagation, the spread of behaviors being best studied under the lens of adoption, and the spread of entities being best studied under the lens of awareness.

Software vulnerabilities could be studied with regards to awareness, indicating the knowledge of vulnerabilities among the developer community, or with regard to adoption, indicating the spread of secure practices. In this study, we focus on awareness and engagement as the indicators of information spread as they are a prerequisite for the adoption of good practices.

#### 2.1.2. Mechanism

In order for information to spread from one user to another, the first user must take some action on the information that causes it to be broadcast in a manner that is visible to the second user. There are many action types or mechanisms by which information can spread from one user to another, and these mechanisms may be constrained by the specific platform under consideration. However, the possible spread mechanisms can be categorized into several common types. These include *posts*, in which the user creates an original post, e.g. tweets on Twitter, repository creation on GitHub, and posts on Reddit; *re-shares*, in which a user rebroadcasts another user’s post, e.g. retweets on Twitter and forks on GitHub; or *responses*, in which a user provides their own commentary on another user’s post, e.g. replies on Twitter and comments on Reddit.

#### 2.1.3 Observable

We must next define an observable that allows us to detect whether information has spread from one user to another. Under the awareness criteria, a post, re-share, or response action by a user will spread the information to all users who view the resulting action. However, this set of users is almost always unobservable. In this study, we leverage a high precision approach to identifying the audience members, which is to focus on the users who interact with the share event of the original user. These interactions, which could include re-shares, responses, likes, etc., provide a clear indication that the user in question has observed the information. Obviously, not all users who observe a post will interact with it. Therefore, this measure of information spread provides a lower bound on the actual number of users who have received the information from the specified user action.

#### 2.1.4 Measure

We apply a comprehensive list of measurements in order to analyze the spread of information. These include measures of core properties of information spread—size, depth, breadth, depth to size ratio, lifetime, unique users, and structural virality that have been successfully used before [14, 15]. We also add two new measurements inspired by their effectiveness in economic experiments to measure income inequality across countries—Gini coefficient [16], Palma ratio [17]. We rely on them to measure discussion disparity or inequality. Size, depth, breadth, structural virality, and unique users have also been used before for analyzing information cascades. We provide definitions of these measurements below using Reddit or Twitter as example platforms.

- *Size* A measure of the scale of information spread. It is a count of all retweets, quoted tweets, and replies to a particular root tweet, including the root tweet for Twitter. On Reddit, the count of all the replies to a post, including the post itself.
- *Depth* Analogous to the depth of tree structures. Measures how deep discussions go in the discussion threads.
- *Breadth* Maximum breadth of any branch of a discussion thread. Measures the longest path of interactions within the discussion thread.
- *Depth to size ratio* Ratio of a discussion thread depth to a discussion thread size. On Twitter, a high depth to size ratio value means that the information spread far beyond the original user’s circle. On Reddit, it indicates a more in-depth discussion about the original topic.
- *Lifetime* A measure of how long a discussion lasted. The difference in time between the original tweet/post and the last comment/retweet in the discussion thread. Unless otherwise stated, we measure lifetime in days throughout the paper.
- *Unique users* The number of unique users who participated in the discussion thread. A measure of the actual number of users who were able to partake of the information.
- *Gini coefficient* Gini coefficient demonstrates the disparity of user participation in discussion threads. Values closer to 1 represent threads where most of the discussions are dominated by a few people while a value of 0 represents threads where each tweet/post/comment is made by a different user.
- *Palma ratio* Palma ratio also demonstrates the disparity of user participation in a discussion thread. Higher values indicate repeated participation by a few users. Even if the discussion thread size is large, if the Palma coefficient is high, the information has not reached many users. The same is true with the Gini coefficient.
- *Structural Virality* Consider two Twitter discussion threads both having a size of 111, including the root. In the first cascade, 110 users retweet the root directly while in the second, ten users retweet the root directly and these ten retweets are subsequently retweeted ten times each. The second thread will have a higher structural virality [18]. High structural virality on Twitter means that the information about a certain vulnerability has reached a wide variety of users rather than a limited group. On Reddit, high structural virality can mean that there was a more in-depth discussion about a vulnerability.

Note, there are more nuanced measures of information spread that focus on evaluating recurrence—renewed bursts of activity for existing information [19], persistent groups—small, committed groups that can influence the rate of spread [20], and cross-platform information spread [21]. All of these measures and more are publicly available [22]. However, in this work we focus on measuring and contrasting core properties of information spread and the network structure across three social platforms.

## 3 Multi-platform social data collection

### 3.1 Software vulnerability data

The National Vulnerability Database (NVD) curates a list of software vulnerabilities and assigns a unique identifier—CVE identifier—to each vulnerability. The database also provides a description for each vulnerability and the date when it was published to the database plus a severity score, as determined by the Common Vulnerability Scoring System (CVSS). The CVE identifier is of the form *CVE-Year-XXXXXX*, where *Year* represents the publication year of the CVE and *X* is a digit. The NVD has been maintained since 1988 and has published more than 100,000 vulnerabilities to date.

### 3.2 Social network data: Twitter, Reddit and GitHub

We created a corpus for analyzing the spread of information about software vulnerabilities by searching for specific software vulnerability mentions—CVE IDs in public archives of Reddit posts and comments [23], tweets obtained through the Twitter API [24], and the full GitHub archive [25]. We accessed all our data in accordance with the terms of service of all three social media platforms. Specifically, we searched for matches to the regular expression:

CVE[-,–][1, 2][0, 9]\d{2}[-,–]\d{4,7}

In this paper, we used data within the time period between January 2015 and September 2017, focusing on major cyber attacks that happened during that period. Our data covers a good number of CVE IDs across the three platforms:

- The total number of CVEs covered across three platforms (Twitter, Reddit and GitHub) is 21,795.
- The total number of CVEs published by NVD between January 2015 and September 2017 is 22,809.
- The total number of CVEs published by NVD till September 2017 is 89,208.

**Fig 2:**
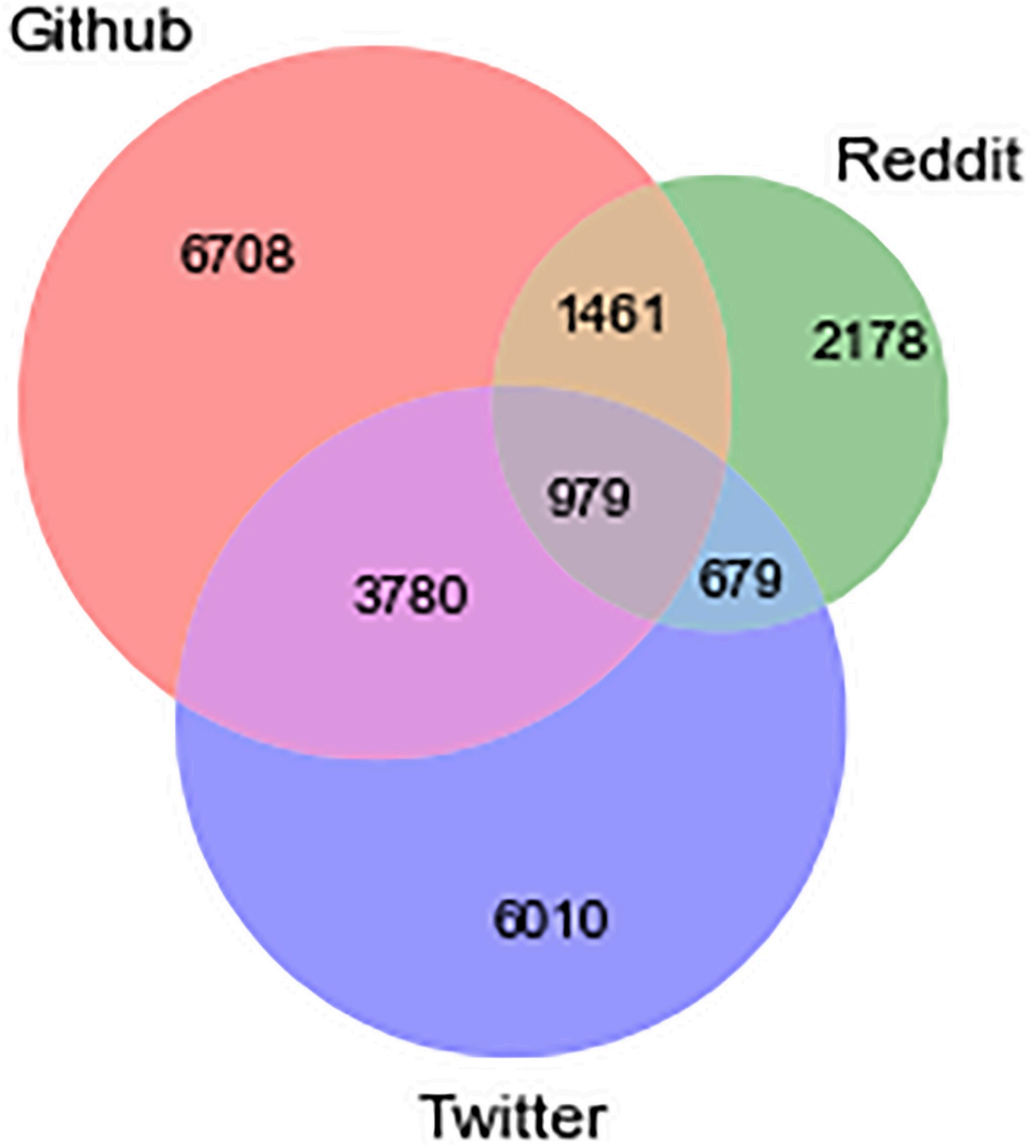
The overlap of software vulnerabilities discussed across the multiple social platforms.

The number of different vulnerabilities discussed in the three platforms is not uniform. Fig 2 shows the number of vulnerabilities for each platform and the overlap between platforms. GitHub has the highest variety of CVE IDs being discussed (12,928) and this makes sense since GitHub is a platform geared towards software development. Even though Twitter is a more general platform, a high number of CVE IDs (11,448) were also discussed on Twitter, out of which 4,798 were also mentioned on GitHub and 1,658 were also talked about on Reddit. Reddit has the lowest total number with only 5,297 CVE IDs being mentioned. Only a small fraction of the total CVE IDs was discussed on all three platforms.

Following our definitions within the information spread framework, we collected the data by focusing on several mechanisms of spread. More specifically, for GitHub, we looked at the counts of the events mentioning a particular CVE identifier. We take the view of GitHub being a collaborative social network [26, 27]. We specifically searched GitHub events with text fields such as *IssuesEvent*, *PushEvent*, and *CommitCommentEvent*. For Twitter, we looked at all tweets, retweets, quoted tweets, and replies. For Reddit, we searched in all posts and comments.

**Fig 3:**
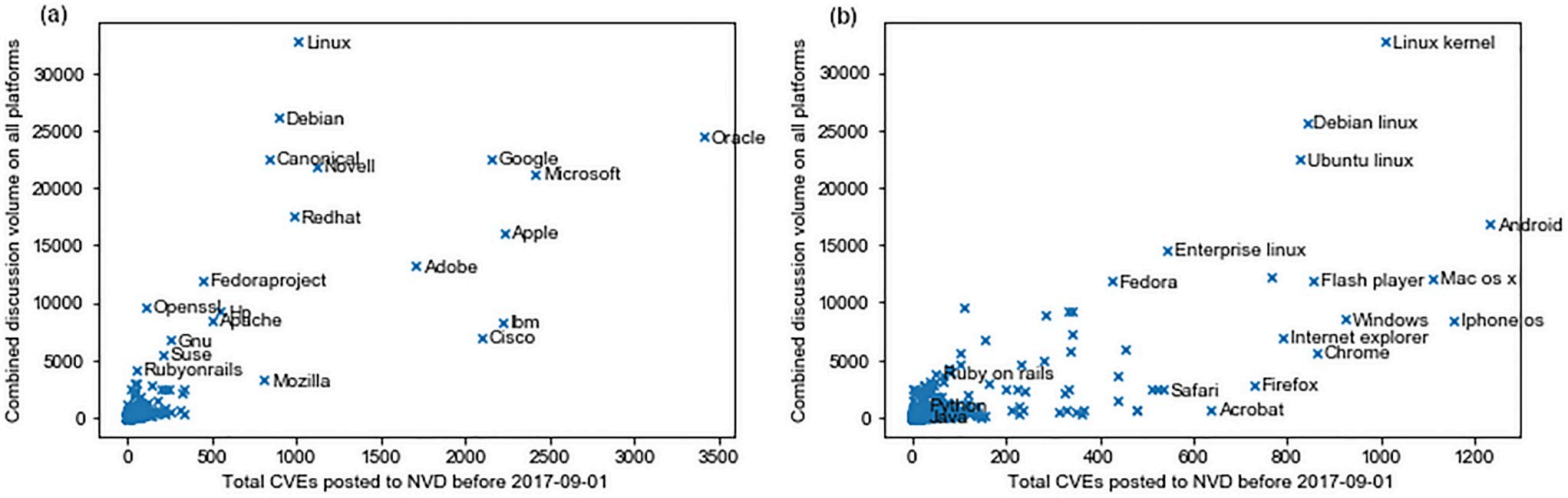
Discussion volume on social media vs vulnerabilities published to NVD for different vendors and products. (a) Vendors (b) Products.

Fig 3 shows the total discussion volume on all three platforms for different vendors and products as a function of the number of vulnerabilities published to NVD until the final date of our data. We can see that for different flavors of Linux (Linux kernel, Debian, Ubuntu, Enterprise Linux, and Fedora), the discussion volume is very high, and for most of them, it is proportional with the number of vulnerabilities in NVD for the product. For Mac OS X and Windows, the discussion volume is not on par with the number of known vulnerabilities. It is even less for browsers such as Safari and Firefox. Although Acrobat has 213 more vulnerabilities published to NVD than Fedora, the total discussion volume for Acrobat is only 728 posts, while that for Fedora is 11,855 posts. Although exact numbers for the user base of Acrobat and Fedora are not available, both are fairly popular and it is safe to say that an attack targeting vulnerabilities in either can have a comparably large impact. As such, it is interesting to see such a disparity in discussion volumes between them.

**Fig 4:**
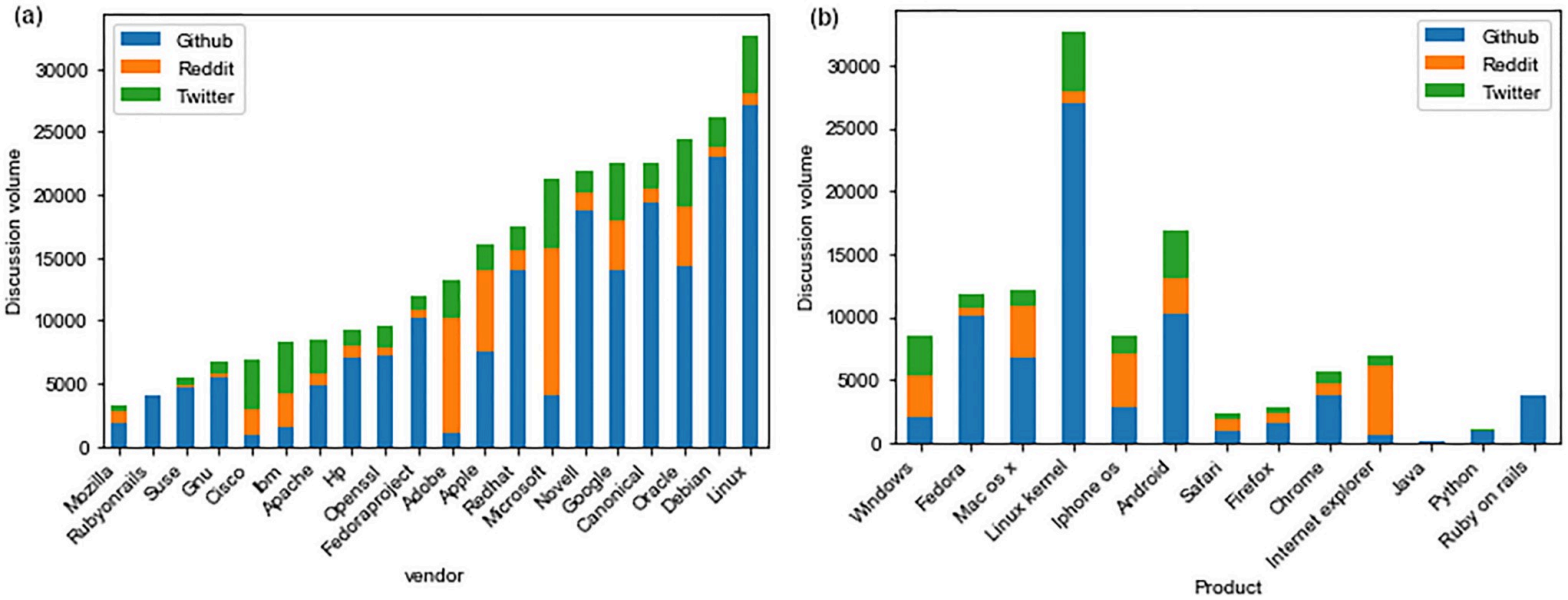
Discussion volume distribution on three social media platforms for different vendors and products. (a) Vendors (b) Products.

Fig 4 shows the distribution of discussion volume across GitHub, Reddit, and Twitter. For most products and vendors, the highest discussion activity is on GitHub. After GitHub, for some vendors and products, the activity is higher on Reddit and for others, it is higher on Twitter. Interestingly, for Windows, the discussion activity is similar on all three platforms, while for Internet Explorer, the most activity is on Reddit. Similarly, iPhone OS vulnerabilities are discussed more on Reddit while for Android, there is more discussion on Android.

## 4 Network topology analysis

Network structure and properties play a role in information diffusion. In this section, we first describe our network construction approach for Twitter, Reddit, and GitHub based on user discussions of software vulnerabilities. We then present the measurements we use for network analysis and present the results of the analysis.

### 4.1 Network reconstruction

GitHub network is an attributed bipartite graph *G* = (*V*, *R*, *E*) where each node is a GitHub user, and there is an edge between *v*_*i*_ and *v*_*j*_ nodes if *v*_*i*_ and *v*_*j*_ mention any CVE IDs in repositories to which they contribute code *r*_*k*_ ∈ *R*. We construct a user-user network based on the bipartite projection on the user node side. All our network measurements are based on this projection and not on the original bipartite network.

Reddit network is an attributed graph *G* = (*V*, *S*, *E*) where each node is a Reddit user and there is an edge between *v*_*i*_ and *v*_*j*_ nodes if and only if *v*_*j*_ is subscribed to the same subreddit *s*_*k*_ ∈ *S* as *v*_*i*_, and *v*_*i*_ comments on posts generated by *v*_*j*_ that mention CVE IDs or vice versa.

Twitter network is an attributed graph *G* = (*V*, *E*), where each node is a Twitter user and there is an edge between *v*_*i*_ and *v*_*j*_ nodes if information with CVE IDs posted by *v*_*i*_ is observed and acted upon using retweet, mention or reply actions by *v*_*j*_.

**Fig 5:**
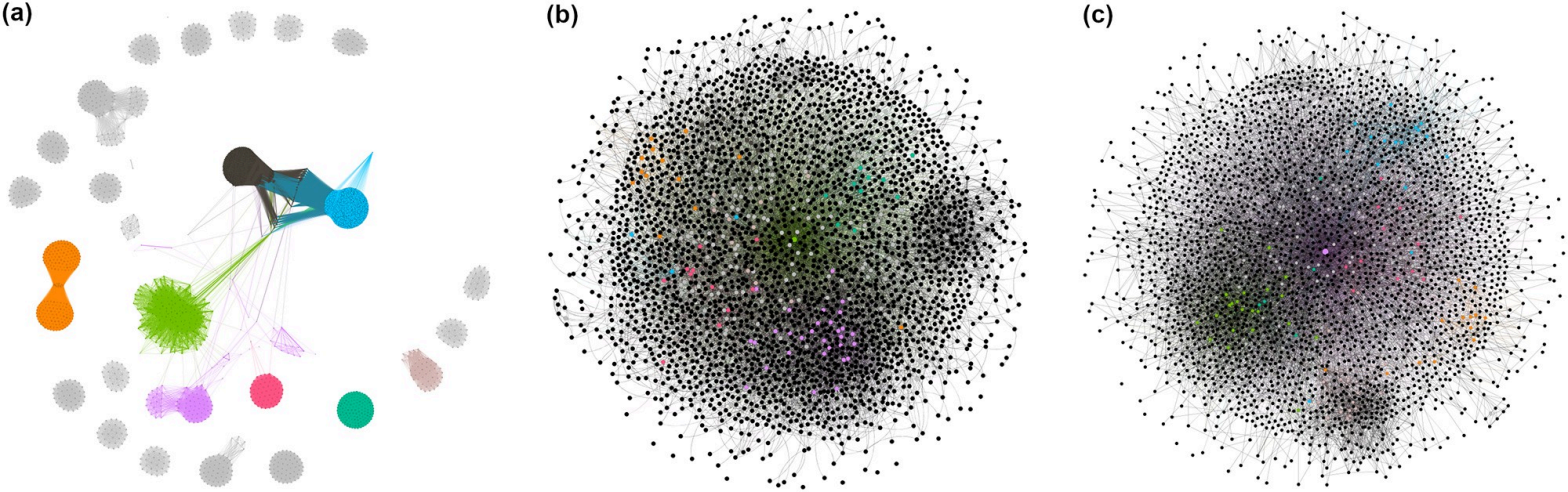
Projected user-user network visualizations for GitHub (degree ≥25), Twitter, and Reddit (degree ≥15). (a) GitHub (b) Twitter (c) Reddit.

Similar to standard practices [28, 29, 30], we treat all networks as undirected and unweighted, and visualize them in Fig 5. In the following discussion, we present a list of measurements we relied on to analyze network topology in our three social platforms.

### 4.2 Network measurements

Below we outline network topology measurements that are know to effect information spread in social networks [28, 29, 31, 32, 33, 30, 34, 35, 14].

*Density* is defined as the degree of connection among individuals in a network, which reflects the overall proportion of connections between individuals [36]. The dense network implies the large number of interconnected nodes, while a sparse network is an open or radial network where users hardly know each other [29].

*Average Shortest Path Length* This quantity measures the average shortest path length between nodes in a graph. A small world graph has a large number of path lengths around the same as the average path length of an equivalent Erdős-Rényi (ER) random graph (same number of nodes and edges as in the original graph but with an ER topology) [37].

*Average Clustering Coefficient* This quantity tells us, on average, what is the ratio of the actual number of triangles in the neighborhood of a node to that of the possible number of triangles. Average clustering coefficient values range from 0 to 1. For small-world graphs, average clustering coefficients are much larger than those for equivalent random graphs [37].

*Assortativity* is the Pearson correlation coefficient evaluated over all pairs of degree values of linked nodes. Assortativity coefficient ranges from –1 to +1. A large positive value of assortativity coefficient signifies that nodes with similar degrees tend to get connected, whereas a large negative value means that high degree nodes tend to connect to low degree nodes [36].

*Modularity* is defined as the fraction of edges that lie in a community for the given graph minus the fraction of edges in the same community for a null model with an equivalent degree distribution. Modularity values range from –1 to +1. Modularity measures how well a network resolves into modules or communities [38, 39]. Networks with large positive modularity values (above 0.4) typically exhibit a high community concentration.

*Degree distribution* This is the distribution of the number of neighbors that each node has in a given graph. In many social graphs, a large number of nodes have a small degree, while a small number of nodes have a disproportionately high degree, signifying the scale-free power-law nature of the degree distributions [40].

#### Twitter network analysis

**Fig 6:**
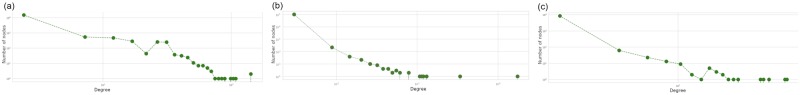
Degree distributions for GitHub, Twitter, and Reddit networks of users who participate in CVE-related discussions. (a) GitHub (b) Twitter (c) Reddit.

As shown in Table 1, the average shortest path length is 5.57. The average clustering coefficient being 0.16 is much greater than the equivalent ER average clustering coefficient (equal to the density). Thus, this network appears to be a small-world network. Further, the network has a power-law degree distribution as shown in Fig 6, with a fairly noticeable negative degree assortativity. This can perhaps be explained by a large number of interactions between high-degree popular users and low-degree followers. We observe that Twitter network has small communities connected by hubs that might act as gatekeepers of information from one community to another. This is in line with previous research that found hubs to have contrasting effects on information diffusion [14].

**Table 1:** Network topology analysis for Twitter, Reddit, and GitHub networks of users discussing CVEs.

Network Measurement	Twitter	GitHub	Reddit
Nodes	8,543	16,856	98,855
Edges	14,255	207,317	213,607
Density, 10^−3^	3.90	1.46	0.04
Average Shortest Path	5.57	4.28	4.26
Assortativity	–0.25	0.24	–0.05
Number of Connected Components	228	9,263	86
Average Clustering Coefficient	0.16	0.37	0.08
Modularity	0.74	0.71	0.74

#### GitHub network analysis

GitHub network is highly connected with the number of edges being close to the number of edges for the Reddit network even though it has only around one-fifth of the nodes in the Reddit network. The average shortest path length is about 4.28 for this network and it appears to be a non-small world network. The clustering coefficient is moderately high (0.37), and so is the assortativity coefficient (0.24) and the modularity (0.71). This network, therefore, appears to be formed by a large number of dense communities—thousands of connected components—connected by long paths (as can also be seen in Fig 5) and the degree distribution appears to show noticeable deviation from the power law. The moderately large positive assortativity is perhaps a consequence of the existence of a number of large cliques.

#### Reddit network analysis

The mean shortest path length is about 4.26. Also, the clustering coefficient is pretty low (0.08) compared to other social networks. Overall, this network exhibits small-world nature to a degree, but that could be a result of many short chain-like structures. In terms of assortativity, the network appears to be fairly neutral. A high modularity value (0.74) encourages information spread. Since users participating in the same subreddit have similar interests, a high modularity value makes sense for Reddit. The low density of Reddit points towards low interaction among users even if they are subscribed to the same subreddit. For there to be an observable information spread on Reddit, users either have to create a post about a vulnerability or comment on a post with a CVE ID. Most users on Reddit are passive browsers and likely do not interact with the posts [41–43]. This makes observable information diffusion much harder in Reddit.

#### Multi-platform network analysis and information spread

As has been shown in the literature, community structure and network assortativity can effect spread patterns and can be good predictors of the strength of diffusion of memes on Twitter [30]. Strong communities enhance social reinforcement [32], and therefore enhance local information diffusion; weak community structure enhances the global spread. Moreover, [29] demonstrated that information spreads further and faster across cluster-lattice networks. GitHub has a higher clustering coefficient, the number of connected components and high assortativity as compared to the other two platforms. This means that on average information around certain vulnerabilities travels faster within GitHub communities compared to Twitter and Reddit. Note, Twitter and GitHub networks are very different as shown in Fig 5 despite displaying similar values, thus their community structure plays a different role in the way vulnerabilities spread. Moreover, high negative assortativity for Twitter indicates that information diffusion is mostly dependent upon the influence of the user creating the tweet. If a user with a high number of followers tweets, it is likely to spread faster and wider. Reddit users do not exhibit tight communities e.g., low clustering coefficient, assortativity and a small number of connected components, thus information spreads slower on average on Reddit.

As has been shown by previous work [34], network modularity also effects information diffusion. Higher network modularity encourages intra-community spread and low modularity encourages inter-community spread. GitHub exhibits strong community structure, high assortativity and high modularity, compared to Twitter and Reddit that facilitates global information diffusion.

Hubs and degree distribution have been studied extensively due to their role in epidemic spread [28]. Hubs however have been shown to have contrasting effects on information diffusion [14]. Although hubs are more likely to generate cascades, large hubs can act as a firewall that actually hinder the cascade continuation, as we observe on Twitter.

Finally, [33] showed that network density is positively related to information diffusion. Twitter network has the highest density which enhances information spread while Reddit has the lowest density.

## 5 Vulnerability spread analysis

In this section, we present an analysis of spread patterns of discussions about software vulnerabilities on GitHub, Twitter, and Reddit. We evaluate the speed and scale of discussion spread about all vulnerabilities, and highlight how the severity and exploitability of a vulnerability affect its spread. We also measure the spread of specific vulnerabilities targeted by state-affiliated advanced persistent groups (APT) [44, 45] or targeting a specific operating system or a product. In addition, we evaluate how attributes of the user who started the discussion thread, for example, if the user is predicted to be a bot or a human, change the rate of spread on Twitter. Finally, we measure how content attributes like post polarity, change the rate of spread on Reddit.

### 5.1 CVE discussion spread across platforms

We found that *discussions about most vulnerabilities usually start on GitHub*. For 46.64% of the vulnerabilities, the discussions started on GitHub before moving to Twitter and Reddit. For 16.14% of the vulnerabilities, these discussions start on GitHub even before they are published to NVD. GitHub also has a security alert system that sources apart from NVD to track vulnerabilities, which might be a reason why vulnerabilities are discussed on GitHub before being published to NVD. Discussions for 26.78% vulnerabilities start on Twitter and for 26.58% vulnerabilities, they start on Reddit. Similarly, 3.26% of the total vulnerabilities in our dataset are discussed on Twitter and 8.75% are discussed on Reddit before being published to NVD.

**Fig 7:**
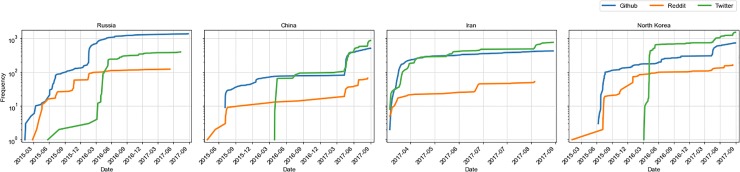
Vulnerability discussion spread across social platforms—GitHub, Twitter, and Reddit for CVEs exploited by country-specific APTs.

**Fig 8:**
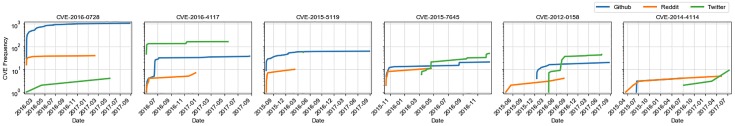
Vulnerability discussion spread across social platforms for CVEs exploited by Russian APT groups (APT27 and APT 29).

Fig 7 shows the cumulative frequency of daily discussions of vulnerabilities in our three social platforms for specific vulnerabilities exploited by country-specific APT groups. We are looking at vulnerabilities exploited by Russia, China, Iran, and North Korea since these are the highest threats to national security. They are also the countries that feature most prominently in APT threat groups that abuse vulnerabilities [45]. There are marked differences in discussions around vulnerabilities exploited by different country’s APTs in the three platforms. For vulnerabilities exploited by Russia, China, and North Korea, there are differences in the time when the discussions start in the three platforms. For all four countries, the discussions of vulnerabilities exploited by their APTs start on GitHub before Twitter, but as soon as it picks up on Twitter, the discussion there increases rapidly and *the discussion volumes between Twitter and GitHub remain quite similar throughout the time period after that for most vulnerabilities*. For most countries except Russia, the volume on Twitter is the largest at the end of our observation time and the volume of Reddit discussions is the smallest. Even for vulnerabilities targeted by Russia, four out of the six vulnerabilities have the largest volume on Twitter as we can see in Fig 8. CVEs targeted by China and North Korea start their spread on Reddit, then GitHub, then finally Twitter. CVEs targeted by Russia start their spread on GitHub. For CVEs targeted by Iran, discussions start at nearly the same time across platforms.

The heatmap shown in Table 2 also demonstrates that the daily CVE discussion volume on GitHub and Twitter are correlated the most compared to other platform combinations: Pearson correlation is 0.492 and Normalized Mutual Information (NMI) is 0.676. The NMI values for GitHub & Reddit and Reddit & Twitter are negative. This could be attributed to the fact that CVE discussion volume on Reddit is inherently low in general, and thus it is hard to establish a correlation between Reddit and other platforms.

**Table 2:** Daily CVE discussion volume correlations across social platforms measured as Pearson correlation and NMI between GitHub events, Reddit comments, and tweets (**significant at *p* < 0.01).

	GitHub & Reddit	GitHub & Twitter	Reddit & Twitter
NMI	0.564	0.676	0.367
Pearson	−0.047	0.492 **	−0.055

### 5.2 Cascading behavior of CVE discussions

In order to measure the scale and speed of discussion spread, we construct discussion threads also known as information cascades. On Twitter, we take the original tweet that mentions a vulnerability as the root of the discussion thread. Any subsequent retweets, quoted tweets, and replies to the root tweet and to any tweet in the discussion thread form the discussion tree. We rely on follower and time information to reconstruct the discussion threads based on time-inferred diffusion as described in [18]. The discussion tree construction on Reddit is simpler. Any post that discusses a vulnerability is the root of the tree and all comments received by that post, including nested comments, become part of the discussion tree [46]. Cascades are tree structures that are also inherently directed graphs with a single directed path from the root node to all other nodes in the graph. Since nodes in a cascade are tweets for Twitter and posts/replies for Reddit, the weight of the edges in the cascades are always 1. Note, the nuance of whether the networks are directed or not is not important to contrast the network structure across three social platforms, however the direction is important to analyze the ways information cascades. Therefore, when we measure how information spreads through cascades the temporal dynamics and the spread direction is important.

There is no straightforward way to create discussion threads on GitHub, so we focused on Twitter and Reddit for this part of the analysis. In total, we reconstructed 5,064 discussion threads for Twitter and had 2,207 discussion threads for Reddit.

Table 3 presents discussion thread measurements for CVE-related discussions on Twitter and Reddit. The format of direct commenting on Reddit encourages more engaging discussions compared to Twitter. Reddit discussion threads are generally larger in size than Twitter, although Twitter discussion threads can also reach a maximum size of 329 tweets. There are large variations in discussion thread sizes in both platforms, although small median sizes (2 and 14, respectively) indicate mostly smaller discussion threads on Twitter and Reddit. On average, Reddit discussion threads are deeper and wider even though the median breadth is also small at 5 on Reddit. Discussion threads in general also last longer on Reddit than on Twitter. On Twitter, maximum lifetime is almost five years (1,761 days), although most of Twitter discussion threads are short-lived—about 9 hours (0.04 days). On Reddit, the maximum lifetime is 178 days, and the median lifetime is 35 hours (1.47 days). This indicates that on average discussions about a certain vulnerability only occurs around the time when that CVE is made public or published to NVD. The highest structural virality on Twitter is 17.61 and that on Reddit is 16.60. The average values are much lower at 1.30 and 3.83, respectively. This can also be attributed to the small discussion thread sizes on both platforms.

**Table 3:** Contrasting measurements of CVE-related discussion spread on Twitter versus Reddit. Average values are higher for Reddit compared to Twitter, but median and maximum values are significantly higher for Twitter.

	Twitter: 5,064 cascades			Reddit: 2,207 cascades		
Measurement	*μ*±*σ*	Median	Max	*μ*±*σ*	Median	Max
Size	4.34 ± 11.34	2.00	329	118.33 ± 473.83	14.00	8662
Depth	1.40 ± 1.46	1.00	51	6.24 ± 5.78	4.00	54
Breath	2.17 ± 4.02	1.00	117	26.94 ± 123.40	5.00	3559
Lifetime (days)	3.02 ± 37.07	0.04	1761.35	9.10 ± 24.58	1.47	178.17
# Unique Users	4.29 ± 11.16	2.00	329	60.72 ± 258.91	9.00	4981
Structural Virality	1.30 ± 0.68	1.00	17.61	3.83 ± 2.56	3.20	16.60

We found that there are some distinct users on Twitter who can cause a certain piece of information to spread far if they tweet about it. These are mainly accounts focused on news about software vulnerabilities such as *FireEye*’s Twitter account, *The Best Linux Blog In the Unixverse*, and *The Hacker News* along with accounts belonging to the experts in the cybersecurity domain.

Any user can participate in a discussion thread multiple times, for example, by retweeting and replying to a tweet on Twitter and by adding multiple comments to the same post on Reddit. On Twitter, the average number of unique users is close to the average size of discussion threads, showing that it is less common for the same users to participate in a discussion twice. The maximum size and the maximum number of unique users exactly match for Twitter, meaning that even for the largest discussion thread of size 329, all of the participating users were unique. However, this is not true for Reddit. The average number of unique users is nearly half the average size of discussion threads for Reddit. A user is very likely to contribute more than once on the same cascade. A discussion thread on Reddit has to be nearly double the size of that on Twitter for information to spread to the same number of users.

### 5.3 Spread of vulnerabilities with different severity

The NVD provides a Common Vulnerability Scoring System (CVSS) score for each vulnerability, which is a combination of *exploitability and impact scores for the vulnerability*. NVD categorizes each vulnerability with a CVSS score between 0.1—3.9 as low severity, between 4.0—6.9 as medium severity, 7.0—8.9 as high severity, and 9.0—10.0 as critical. We analyzed whether CVE discussions spread differently for these four categories. *The highest number of discussions were related to vulnerabilities with high severity, and those with low severity had the smallest number of discussion*.

**Fig 9:**
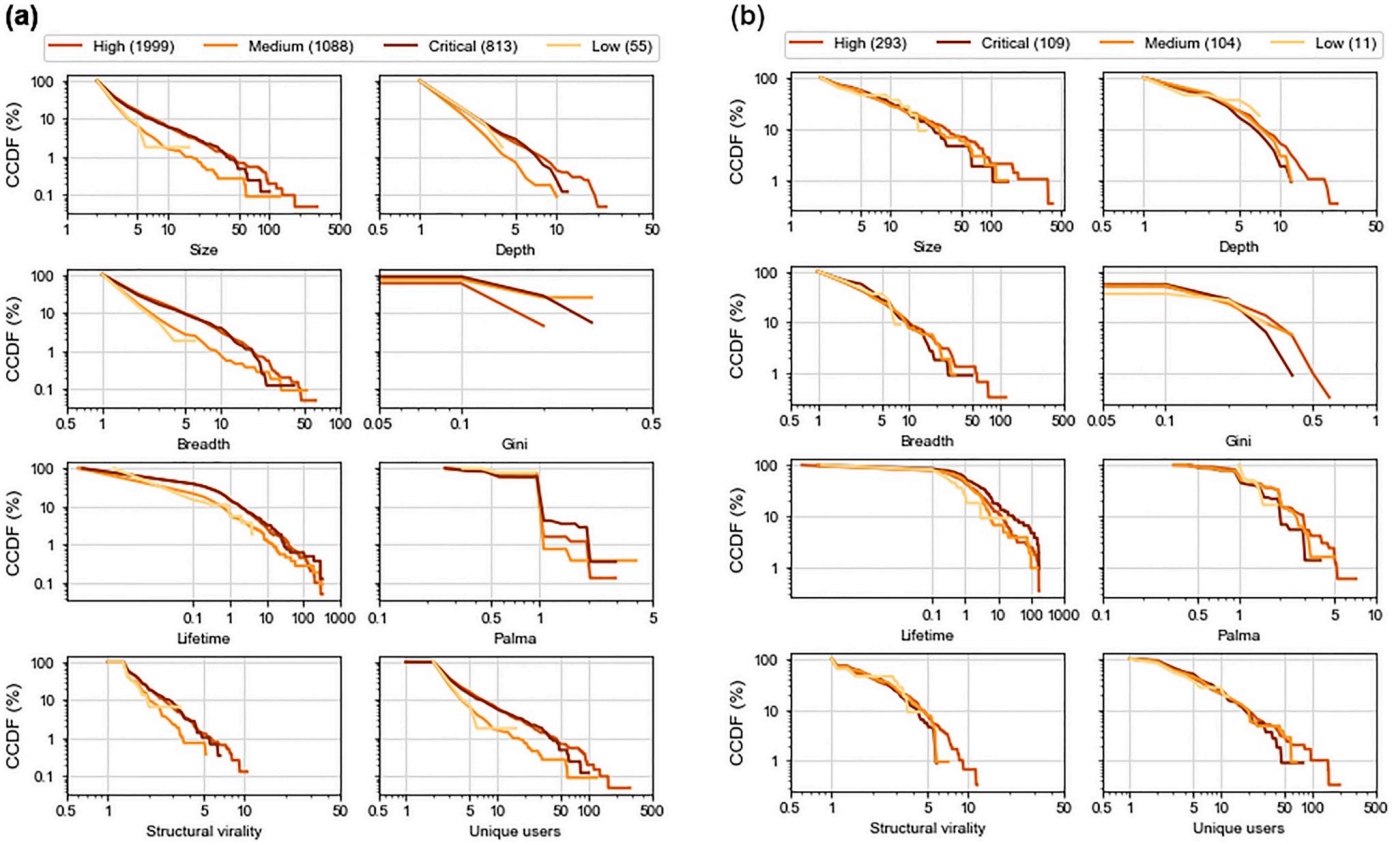
CCDF plots of information spread measurements of vulnerabilities with different severity on Reddit and Twitter. The numbers in parentheses are the number of discussion threads. Plots are shown in a log scale for both axes. (a) Twitter (b) Reddit.

Fig 9 shows a complementary cumulative distribution function (CCDF) plots of nine information spread measurements of discussion threads. Since there are a different number of discussion for each level of severity, it makes more sense to compare the percentage distributions, which are plotted in the figures. To illustrate how these CCDF plots present the measurement data, we can look at the depth plot for Twitter. The line plot for medium severity starts at a depth value of 1 and a 100% CCDF value, meaning that all discussion threads related to a medium severity vulnerability have a depth of at least 1. The line terminates at a depth value of 10 and a CCDF value of 0.1, meaning that only a fraction equal to 0.1% of those threads reach depth 10.

From Fig 9a, we can see that a higher proportion of discussion threads where the root tweet discusses high and critical vulnerabilities reach larger sizes, depth, and breadth on Twitter. *Information about vulnerabilities that are high severity spreads to a higher number of users and gets shared more*. Tweets discussing high severity vulnerabilities seem to spread more than those discussing critical vulnerabilities but the difference is not significant. For all measurement values, discussions mentioning vulnerabilities with high severity are significantly larger, deeper, wider, last longer and get more user participation than discussions mentioning medium vulnerabilities (*p* < 0.001). (Unless otherwise mentioned, we have used Mann-Whitney U as the test of statistical significance throughout this work. We have mentioned p-values for all statistically significant findings.) The same is the case when we compare critical and medium severity vulnerabilities. The differences in lifetime between less severe and more severe posts are not that apparent, but still, tweets discussing low severity vulnerabilities live shorter than those discussing critical (*p* < 0.05) and high (*p* < 0.05) severity vulnerabilities. Similarly, a higher proportion of discussion threads with more unique users for high and critical vulnerabilities, also demonstrates that not only does information about these vulnerabilities gets spread more, but also that information spreads to a larger audience. There are more discussion threads with higher values of structural virality for severe vulnerabilities, indicating that discussions about high and critical CVEs spread to a wide variety of users and are not confined to particular cliques.

Severity score plots for Reddit in Fig 9b show similar trends. The high severity vulnerabilities have the highest number of discussion threads while the lowest number of discussion threads are for low-severity CVEs. From the figure, we can see that discussion threads where the root post talks about a vulnerability with high severity are larger, deeper, and wider in Reddit as well, although the differences are not significant. Discussions about critical posts last the longest as we can see from the plot for lifetime. Posts mentioning critical severity vulnerabilities have longer discussions than those mentioning high (*p* < 0.05), medium (*p* < 0.05), and low (*p* < 0.05) severity vulnerabilities. The plots for the number of unique users as well as for the Gini and Palma scores indicate that the information about highly severe vulnerabilities spreads to a large audience since more unique users seem to participate in these discussions.

### 5.4 Spread of vulnerabilities targeted by APT groups

**Fig 10:**
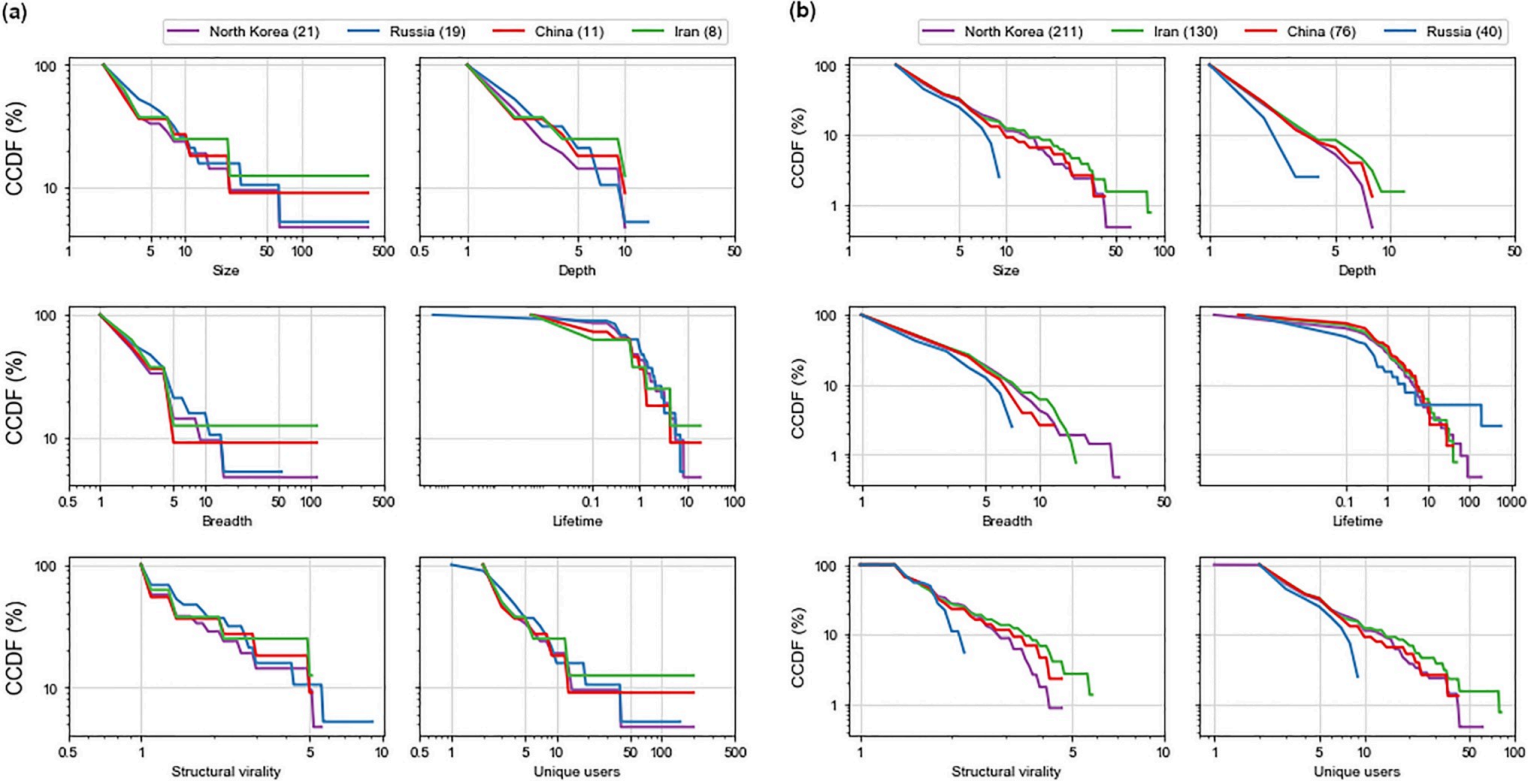
CCDF plots of information spread measurements on Reddit and Twitter for a subset of vulnerabilities exploited by different APT groups. (a) Reddit (b) Twitter.

We took a closer look at the discussion threads that talk about a group of vulnerabilities targeted by known APT groups associated with different countries. From Fig 10 we can see that the most discussed vulnerabilities are those exploited by North Korean APT groups. On Reddit, those exploited by Russia come second while on Twitter, vulnerabilities exploited by Iran are the second most prominent. Although cascades related to all countries reach similar sizes on Reddit, a higher proportion of the discussion threads with vulnerabilities exploited by Iran reach larger sizes, depths, breadths, and also have a higher number of unique users. Discussion threads mentioning vulnerabilities exploited by Russian APT groups obtain the maximum depth and structural virality. A higher proportion of discussion threads for vulnerabilities exploited by Iranian APT groups again have larger sizes, depths, breadths, and unique users on Twitter as well. The cascades for vulnerabilities exploited by Russian APT groups, on the other hand, seem to have smaller sizes and reach shorter depths, but interestingly, they also have the longest lifetime. *On Twitter, discussions about vulnerabilities exploited by Russian APTs last longer than discussions about vulnerabilities exploited by all Chinese (p < 0.01), Iranian (p < 0.01), and North Korean (p < 0.05) APTs*.

### 5.5 Spread of vulnerabilities targeting operating systems

**Fig 11:**
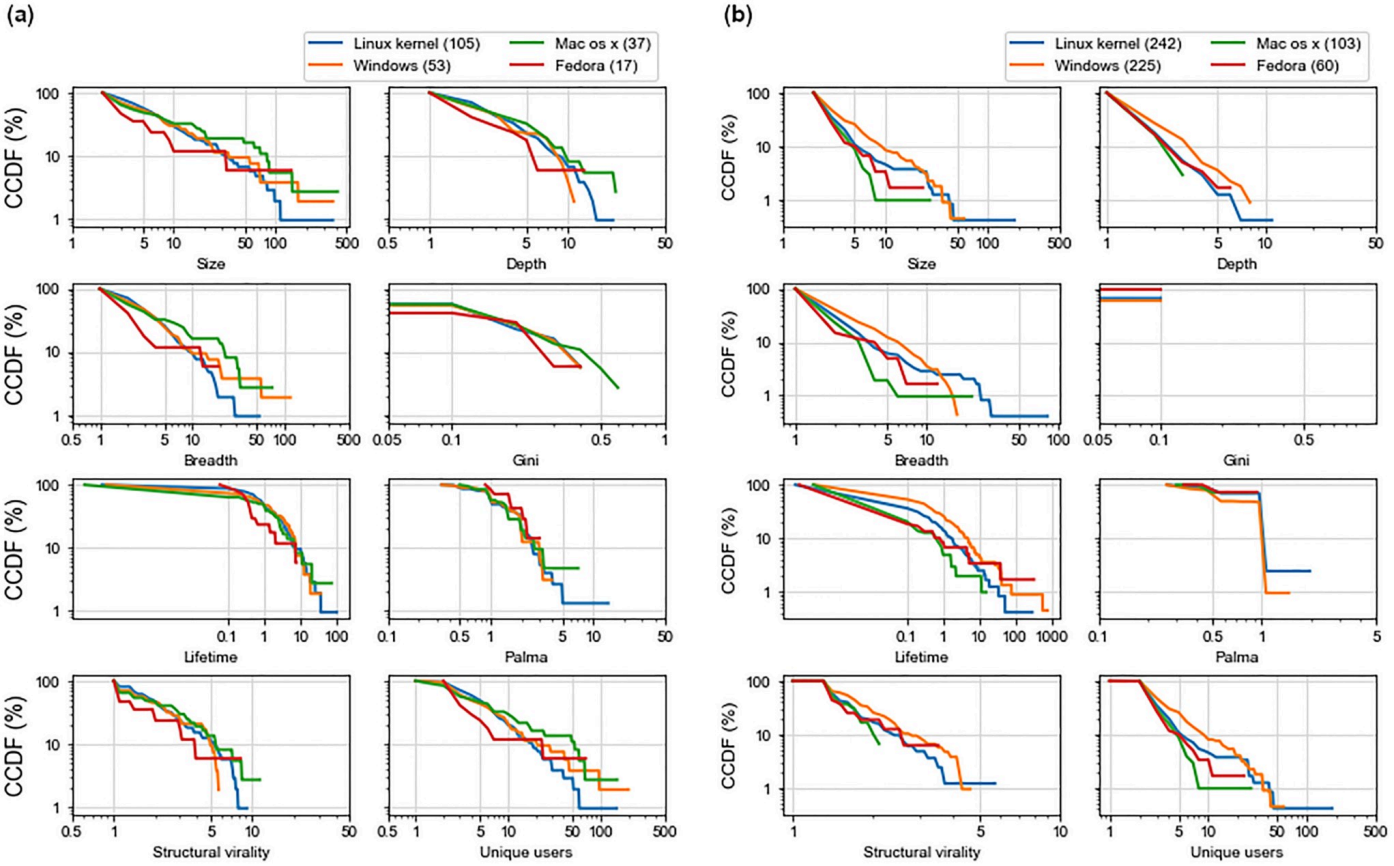
CCDF plots of information spread measurements on Reddit and Twitter for different Operating Systems (OS). (a) Reddit (b) Twitter.

Fig 11 shows the cascade plots for discussion spread of vulnerabilities in Reddit and Twitter for four Operating Systems (OS): Linux Kernel, Windows, Mac OS X, and Fedora. We can see that Linux Kernel has the highest number of discussion threads. However, the discussion threads for vulnerabilities in Windows seem to be the most distinct in terms of measurements from the cascades of other operating systems, especially on Twitter. Although the discussion about a Linux Kernel vulnerabilities has the maximum size, depth, and breadth, *the discussion threads for vulnerabilities on Windows are in general much larger, wider, and deeper*. They not only have larger sizes than those for Linux Kernel (*p* < 0.001), but they also reach more people (*p* < 0.001). The discussion also go deeper (*p* < 0.001) and live for a longer time (*p* < 0.001). This is true when we compare discussion threads of Windows to those of all other operating systems. The discussion threads about Linux Kernel do live longer than those about Fedora (*p* < 0.05). On Reddit, however, the discussions are more clumped together indicating that the spread on Reddit for different products is not very distinct from each other. The most difference in discussion thread measurements is between Linux Kernel and Fedora. The discussions for Linux Kernel vulnerabilities have larger sizes (*p* < 0.05), depths (*p* < 0.05), breaths (*p* < 0.001), unique users (*p* < 0.05), and lifetime (*p* < 0.05) as compared to those for vulnerabilities in Fedora.

Note, CCDFs are normalized measures that account for skewed distributions; however, they do not account for sample uncertainty. There are possible ways to compensate for biases introduced by external factors (i.e. population of different OS). These include but not limited to using bootstrap sampling or report relative differences to the norm.

### 5.6 Additional Twitter analysis: How social bots change the rate of vulnerability spread?

The prevalence of bot accounts on Twitter trying to spread their own agenda is well-known [47–49]. A bot account might try to spread fear in the general population by over-emphasizing the scale and impact of some vulnerability. On the other hand, for awareness raising posts to attract attention, the message should be relayed in a compelling way and not appear glaringly bot-like. We categorized users as likely bots or humans, by using the Botometer tool, which uses a wide variety of user-based, friend, social network, temporal, and content-based features to perform bot vs. human classification [50]. The creators of the tool used a dataset of 15K bot accounts and 16K human accounts collected using the honeypot method [51], and also manually annotated 3,000 users. In their experiments, the tool was able to obtain a very high AUC of 0.95.

**Fig 12:**
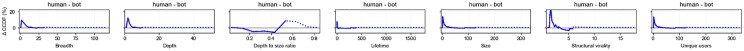
Differences in information spread for root tweets posted by users predicted as humans versus bots. Differences are taken between CCDFs for humans versus bots. Spikes above 0 demonstrate larger numbers for humans. Spikes below 0 demonstrate larger numbers for bots.

In Fig 12, we analyze how information about vulnerabilities spreads when it is tweeted out by a user predicted to be a bot versus when it is tweeted by a user predicted to be a human. We discovered a disproportionately high number of discussions started by human users in our dataset. 4,853 discussion threads were started by humans, while only 168 discussions were started by bots. In order to clearly see the difference between them, we plotted a difference in CCDF values. We can see that there is a higher percentage of discussions started by humans having a size above two. *Discussions started by human-like users grow to significantly larger sizes* (Mann-Whitney U test, *p* < 0.001). Discussions started by human-like users reach up to 329 while those by bot-like users only grow to a maximum size of 127. We show this in the plot by using a solid line for sizes reached by both types of discussions (indicating the difference in their CCDF values) and with a dotted line for the sizes reached only by one of the types of discussions (indicating values for a single type). A large proportion of discussions started by bots (77%) do not grow beyond the size of two, meaning that only one other user retweets or replies to the tweet from a bot. Discussions started by human-like users also reach larger breadths than those by bots (*p* < 0.001). We can see a similar plot for depth as well and discussions started by bots only reach depths of 10 while those by humans reach a maximum depth of 51. In general, discussions by human-like users go significantly deeper (*p* < 0.001). We can clearly see that *discussions started by users predicted to be humans reach larger sizes, depths, and breadths than bot users*.

When we consider the depth to size ratio of discussion threads, the numbers are not always positive as in the case of size, depth, and breath measurements. We can see that a higher proportion of discussions started by bots have a depth to size ratio between 0.13 and 0.43. Although larger values of depth to size ratio are again achieved by discussions started by humans, the difference is not significant. When we look at discussion lifetimes, *only a small proportion of the cascades from both bots (32%) and humans (39%) live beyond their first hour*. Even though discussions started by humans do have significantly longer lifetimes (*p* < 0.01), only 8% of the cascades by humans and 5% of the cascades by bots have a lifetime longer than 3 hours. The results for structural virality and the number of unique users also follow similar patterns to size, depth, and breadth. There is a higher proportion of discussions started by humans with high structural virality (*p* < 0.001). Since unique user counts are highly dependent on discussion thread size, it is intuitive that discussions started by humans have a higher proportion of unique users with larger values (*p* < 0.001).

In many cases, tweets from bot-like users are repetitive and follow a certain pattern, for example, a generic message followed by a URL. Table 4 shows a few examples of tweets by a bot-like user. These tweets each announce a vulnerability, all following the same particular pattern: first the severity, the CVE id, short description of the vulnerability, a link to learn more about it and then the same four hashtags. Although these tweets might have been posted to raise awareness, they are too bot-like and will likely get ignored. On the other hand, the tweets from human-like users are not generic and are likely to capture attention and be more effective at spreading awareness about software vulnerabilities.

**Table 4:** Examples of tweets with vulnerabilities spread by users predicted to be bots versus humans.

Bot	High CVE-2017-8890: Linux Linux kernel https://t.co/OIrRZRQDYs#CVE#InfoSec#Vulnerability#cybersecurity
	Medium CVE-2017-9058: Ytnef project Ytnef https://t.co/8lYRLvBP0M#CVE#InfoSec#Vulnerability#cybersecurity
	Medium CVE-2017-14702: Ersdata Ers data system https://t.co/vnYDhHIBHc#CVE#InfoSec#Vulnerability#cybersecurity
Human	Almost all WordPress websites could be taken down due to unpatched CVE-2018-6389 DoS flaw
	CVE-2017-5638 means Equifax did not patch S2-045 released in March

### 5.7 Additional Reddit analysis: How root post subjectivity and polarity change the rate of vulnerability spread?

As has been previously shown, emotions play significant role in how social interactions evolve and the way information spreads online [35]. To verify whether software vulnerabilities exhibit similar diffusion patterns as other types on information e.g., topics, hashtags etc., we aim to evaluate what role subjectivity of a post plays in the degree of its spread. We want to analyze if users engage more with posts that simply state the facts or with those that also include the poster’s viewpoints towards certain vulnerability mentions and cybersecurity incidents. Malicious actors may use negative or emotionally charged language to increase the hype and seed a fear around certain software vulnerabilities. Also, the most effective way of ensuring that software vulnerabilities are not being exploited is to spread information about them and about any software patches released in order for them to be fixed in codebases. For this reason, it is important to know what kind of language can encourage or discourage information diffusion about vulnerabilities. To distinguish between subjective vs. neutral posts, we assessed the polarity of Reddit posts (positive, neutral, or negative) by using a publicly available tool TextBlob [52]. The tool outputs the polarity score, which is a float within the range from –1.0 to 1.0. We grouped posts with negative polarity values as negative, 0 as neutral, and positive polarity values as positive. In total, we found 395 positive, 188 negative, and 92 neutral posts in our dataset that start discussion threads.

**Fig 13:**
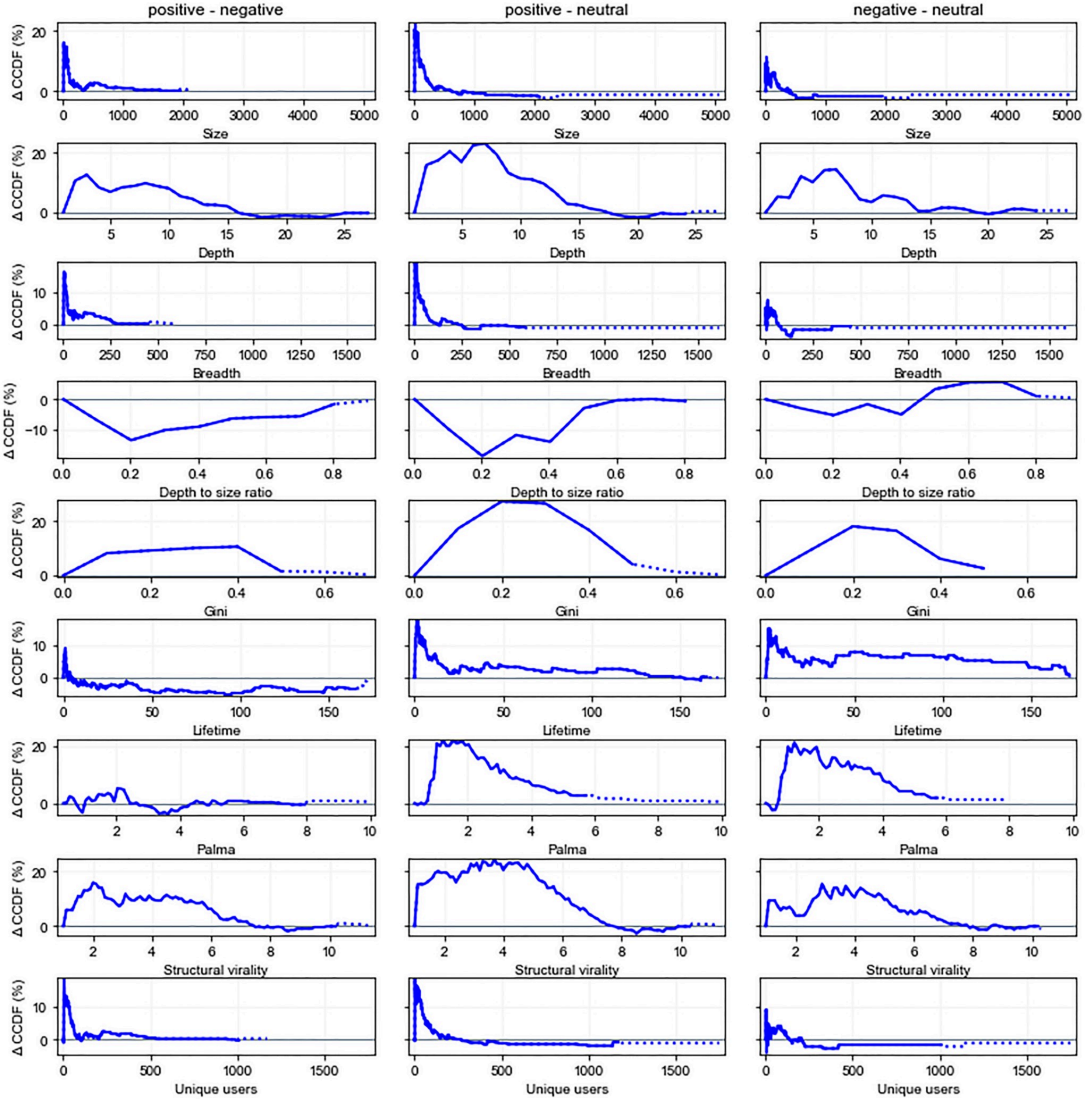
Differences in vulnerability spread for Reddit posts that are subjective (positive, negative polarity) vs. neutral. Differences are taken between CCDFs for all polarity combinations.

Fig 13 shows that *Reddit posts that are subjective with positive polarity reach larger sizes, depths, and breadths compared to negative and neutral posts*. Positive posts are in general significantly larger than negative (*p* < 0.001) and neutral posts (*p* < 0.001). The median size of discussions started by a positive post is 21, that of a negative post is 16 and for a neutral post, it is 12, although a discussion of a neutral post reaches the largest size of 5067. Similarly, positive posts also go deeper than negative (*p* < 0.05) and neutral (*p* < 0.001) posts as well as wider than negative (*p* < 0.001) and neutral (*p* < 0.001) posts. Positive posts around vulnerabilities seem to encourage more discussion than negative or neutral posts. We can clearly see that more positive posts receive higher exposure when it comes to posts about vulnerabilities in contrast to the the findings in [35], where they deal with Twitter posts about a social movement and found that cascades not having a positive message actually tend to be larger in size. This is good news in the case of software vulnerabilities because as we can see from the example subjective posts with positive polarity shown below, they are mostly talking about patches for vulnerabilities:

- PSA: New ransomware campaign (Petya/GoldenEye) being conducted that will render your computer unbootable if infected. Make sure your machines are patched and updated to avoid infection.
- Linux Kernel exploit (CVE-2016-0728): Update ASAP

On the other hand, positive posts have a smaller depth to size ratio than negative (*p* < 0.001), showing that for similar size discussion threads, *negative posts’ discussion reach larger depths*. This presence of more nested comment threads for negative posts might be indicative of the topic being controversial. *Negative posts also have higher structural virality than neutral posts*(*p* < 0.05), meaning that they are more spread out and there is much a more in-depth conversation going on for these posts than for neutral posts. Nonetheless, again positive posts have higher structural virality than both negative (*p* < 0.001) and neutral posts (*p* < 0.001).

When we look at the lifetimes of the discussion threads, there is not a significant difference between the lifetimes of positive and negative cascades. The average lifetimes of positive and negative cascades are 12.75 days and 18.43 days respectively, while the median lifetimes are 2.07 days and 1.91 days respectively. Neutral cascades though have shorter lifetimes than posts that show some emotion i.e. positive (*p* < 0.01) and negative (*p* < 0.05) cascades. When we look at the actual users who participate in cascades, positive posts’ cascades again have a much higher number of unique users than negative (*p* < 0.001) and neutral (*p* < 0.001) posts’ cascades and thus more inequality when we look at the Gini and Palma score plots. This indicates that not only are positive posts’ cascades larger in size, they also reach a larger number of users.

**Fig 14:**
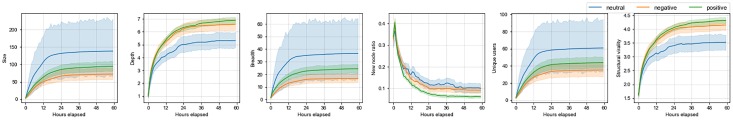
Speed of vulnerability discussion spread on Reddit across cascades started by posts with different polarities.

For these positive, negative, and neutral posts, we also looked at the speed of information diffusion about vulnerabilities on Reddit. The area plot in Fig 14 shows the mean value and confidence interval of cascade measurements for the first two and a half days after the root post. We can see that the cascades started by neutral posts reach higher mean sizes quicker than positive and negative post cascades. Often, discussions about vulnerabilities reach peak sizes and then plateau after the first day of the post irrespective of the polarity of the post. The depth of the cascades also reach close to the maximum within the first two days, but there is still a slow but steady rise as more time goes by, especially for positive posts’ cascades. We can also see that the structural virality for positive and negative cascades increase fast and reach higher values.

We also looked at the ratio of new comments being added at each timestep. We can see that the new node ratio spikes within the first few hours and then decreases steadily. By the second day, only a small number of cascades see new comments. When we look at unique users, we can see that after the first day, we do not see much new user participation. The plot for size and for unique users both indicate that on Reddit, information spread happens mostly on the day of the post, after which the interest completely wanes. We can also see that the 95% confidence interval is very high for neutral posts since the number of neutral posts is the lowest and the standard deviation is the highest. The new node ratio plots are closely clumped together and the 95% confidence interval is also small, indicating that the ratio of new nodes being added each day does not vary much between any types of cascades.

## 6 Discussion and key findings

Our analysis provides empirical evidence of the benefits of relying on social media signals to maintain the awareness, anticipate and prioritize vulnerabilities to watch and fix first. We not only demonstrated that some vulnerabilities are discussed on social media before they are made public on NVD, but also analyzed how useful actionable software vulnerabilities signals are across social platforms. We found GitHub is the most useful platform, but due to the lack of public API, actionable insights can still be gathered from Twitter and Reddit.

User interaction network topology is directly related to the way information spreads within this network. Our contrastive analysis of network topologies across social platforms revealed a few interesting findings. We found that discussions about software vulnerabilities are often driven by popular users or super-spreaders (e.g., nodes with high degree). All three networks analyzed in this work revealed strong community structure and modularity which encourages information spread. However, we demonstrated that the network structure and, therefore, the spread patterns on GitHub are different compared to Twitter and Reddit.

Our novel findings on the relationships between spread patterns and characteristics of vulnerabilities provide novel and actionable insights for cybersecurity analysts towards the awareness in this domain. More specifically, we found that:

- Discussions about vulnerabilities mostly start on on GitHub (46% of discussions), and in 16.14% of the cases, even before the vulnerabilities are published to NVD. Since GitHub is geared mainly towards developers, it makes sense that they are the first ones to be on the lookout for any new vulnerabilities and be the first ones to discuss them. GitHub seems to be a good resource to be in the know about new vulnerabilities. Even though Twitter and Reddit are more general purpose social media platforms, they also have a substantial amount of discussion of vulnerabilities that can be leveraged by cyber analysts.
- GitHub user networks also have higher average clustering coefficient, assortativity and the number of connected components compared to the other two platforms. On GitHub there are a large number of dense communities. Awareness about vulnerabilities is more likely to spread faster on GitHub within the communities, and it is likely that users within certain communities only care about certain vulnerabilities, e.g., vulnerabilities in their programming language of choice and in the libraries they use.
- For most discussions of vulnerabilities exploited by Russian, Chinese, Iranian, and North Korean APTs, the discussions again start on GitHub before Twitter but at the end of our observation time, there are similar volumes of discussion on Twitter and GitHub. The discussion volume on Twitter picks up more rapidly probably because the nature of Twitter is more conducive to the sharing of information than GitHub. If there is a need to encourage awareness about certain vulnerability, Twitter seems to be the likely platform to use.
- Highly severe vulnerabilities produce the most discussions on both Twitter and Reddit platforms and the information about vulnerabilities that are high severity also spreads to a higher number of users and gets shared more. On one hand, it can help raise awareness about these high severity vulnerabilities so that developers can resolve them in their codebases. On the other hand, it also informs malicious actors about the existence of these vulnerabilities.
- There are marked differences in the volume of discussion around different software products. The highest discussion volume seems to be for the different types of Linux. While looking at the spread analysis though, discussion threads for Windows vulnerabilities are much larger, wider, and deeper on Twitter. The Twitter crowd seems to care more about Windows vulnerabilities while the Reddit crowd does not differ much in their discussions of vulnerabilities relating to different operating systems.
- On Twitter discussion threads started by users predicted to be humans reach larger sizes, depths, and breadth compared to those started by users predicted to be bots. These bot-like users in our dataset are usually non-malicious and are mostly accounts set up to make developers aware of vulnerabilities in different operating systems or libraries but the message might not be getting across because the wording is too bot-like.
- On Reddit, discussion threads of positive posts are larger, wider, and deeper than negative or neutral posts. Posts having a positive message and those talking about patches to fix vulnerabilities get more attention on Reddit. Reddit might be a good source of information for users to learn about these important patches and for the owners of the vulnerable codebases to spread information about new fixes for the vulnerabilities.

## 7 Related work

There has been extensive research on how information diffusion occurs on social platforms. Previous works have looked at the spread of text, URLs, and images on Twitter [15, 53, 54] and on Reddit [55–57]. There have also been studies that look into using Twitter to predict the exploitability of software vulnerabilities in order to generate advance warnings [58–60]. On GitHub networks, the information is the code in a repository itself and there have been studies into how these repositories are spread and what factors decide which repositories become popular [61]. Previous research has also noted the delay between vulnerability announcement and publication to NVD [62]. Their vulnerability knowledge base and alert system build from GitHub issues and bugs also proves GitHub to be a rich source of information about vulnerabilities.

Apart from Twitter, Reddit, and GitHub platforms, there has also been research regarding image reshare cascades on Facebook [63, 64] and Pinterest [65, 66] as well as information propagation in the cascades of blog posts on Blogosphere [67]. All of these works look at the properties of cascades or social networks and also at trying to figure out what drives the spread.

In the same vein, there has also been growing research on predicting cascade growth [64, 68, 69], predicting attributes of the root using cascade properties [70], and estimating properties of an incomplete cascade [71]. Researchers have even looked at finding out if the same content is likely to create reshare cascades time and again [72].

While the focus of these previous works has been on characterizing and modeling the cascades, our main focus is on how the same unit of information spreads in multiple social environments. To the best of our knowledge, this is the first work of its kind. Apart from analyzing and measuring discussion spread on Twitter, Reddit, and GitHub individually, we also compare and contrast across these different platforms, providing a better, well-rounded picture of how information about software vulnerabilities truly spreads in online social environments.

## 8 Conclusions and future work

In this work, we presented an in-depth contrastive analysis of discussion spread about software vulnerabilities in three social platforms—GitHub, Twitter, and Reddit. We defined a fundamental information spread evaluation framework that clearly defines the spread mechanisms and observables across platforms, the units of information, and the groups of measurements that can be applied. We were able to show that the information spread behavior is inherently different across social environments in many aspects, including the scale and timeline of discussions as well as the actual number of vulnerabilities being mentioned. We also discussed how several user-level and content-level factors, as well as the network topology influences the rate of CVE discussion spread.

The results of our analysis clearly demonstrate that social platforms reveal actionable signals for software vulnerability awareness. The fact that most CVE discussions start on GitHub not only before Twitter and Reddit, but even before a vulnerability is officially published, is critical knowledge for cyber analysts. These and other findings can be used by analysts as well as by product vendors and codebase owners to increase the awareness about vulnerabilities and software patches among developers as well as general users. Moreover, our multi-dimensional analysis is intended inform and advance a variety of analytical frameworks [73, 14] for simulating information spread across multiple social environments including but not limited to disinformation.

In this work, we looked at the factors that encourage the spread of information and in the future, we want to extend our analysis to how information evolves as it spreads. When people share information, they tend to append their own thoughts and opinions with it. In the cybersecurity domain, a piece of information can be easily modified to spread fear in the general public. An in-depth analysis of how information evolves and the actors that significantly modify the information, the modification strategies they apply to reach a desired outcome will be a crucial future step.
